# Bioactive Compound Profiling of Olive Fruit: The Contribution of Genotype

**DOI:** 10.3390/antiox11040672

**Published:** 2022-03-30

**Authors:** Soraya Mousavi, Vitale Stanzione, Roberto Mariotti, Valerio Mastio, Aristotelis Azariadis, Valentina Passeri, Maria Cristina Valeri, Luciana Baldoni, Marina Bufacchi

**Affiliations:** 1 Institute of Biosciences and Bioresources, National Research Council, 06128 Perugia, Italy; roberto.mariotti@ibbr.cnr.it (R.M.); mariacristinavaleri.mcv@gmail.com (M.C.V.); luciana.baldoni@ibbr.cnr.it (L.B.); 2 Institute for Agricultural and Forest Systems of the Mediterranean, National Research Council, 06128 Perugia, Italy; vitale.stanzione@cnr.it (V.S.); valentina.passeri@isafom.cnr.it (V.P.); marina.bufacchi@cnr.it (M.B.); 3 Estación Experimental Agropecuaria San Juan, Instituto Nacional de Tecnología Agropecuaria (INTA), Consejo Nacional de Investigaciones Científicas y Técnicas (CONICET), Ing. Marcos Zalazar (Calle 11) y Vidart, Villa Aberastain, Pocito, San Juan 5427, Argentina; mastio.valerio@inta.gob.ar; 4 Department of Horticultural Genetics and Biotechnology, Mediterranean Agronomic Institute of Chania, 73100 Chania, Greece; aristoteles@agro.au.dk

**Keywords:** antioxidant, oleuropein, oleocanthal, verbascoside, α-tocopherol, squalene, sterols, olive fruit, olive cultivars, genotype effect

## Abstract

The health, therapeutic, and organoleptic characteristics of olive oil depend on functional bioactive compounds, such as phenols, tocopherols, squalene, and sterols. Genotype plays a key role in the diversity and concentration of secondary compounds peculiar to olive. In this study, the most important bioactive compounds of olive fruit were studied in numerous international olive cultivars during two consecutive seasons. A large variability was measured for each studied metabolite in all 61 olive cultivars. Total phenol content varied on a scale of 1–10 (3831–39,252 mg kg^−1^) in the studied cultivars. Squalene values fluctuated over an even wider range (1–15), with values of 274 to 4351 mg kg^−1^. Total sterols ranged from 119 to 969 mg kg^−1^, and total tocopherols varied from 135 to 579 mg kg^−1^ in fruit pulp. In the present study, the linkage among the most important quality traits highlighted the scarcity of cultivars with high content of at least three traits together. This work provided sound information on the fruit metabolite profile of a wide range of cultivars, which will facilitate the studies on the genomic regulation of plant metabolites and development of new olive genotypes through genomics-assisted breeding.

## 1. Introduction

Olive (*Olea europaea* subsp. *europaea* var. *europaea*), one of the most important oil crops in the world, is particularly important for its high contents of minor compounds, which are mainly responsible for its beneficial health effects and valuable antioxidant activity, making olive oil the healthiest among all vegetable oils [1,2,3]. Olive oil quality is usually measured via fatty acid, polyphenol, phytosterol, tocopherol, and squalene content [4,5,6,7]. The nutraceutical compounds studied in olive oil or its direct precursors are present in the olive fruit pulp, often at higher concentrations. Thus, the olive bioactive metabolites evaluated directly in the fruit pulp may allow the quick screening of a large number of genotypes, providing information on their potential for high-valued olive oil production [8].

*O. europaea* phenols include different subclasses of compounds. Among these, the secoridoids include oleacein (3,4-DHPEA-EDA); oleuropein aglycon (3,4-DHPEA-EA); oleocanthal (p-HPEA-EDA); and ligstroside aglycon (p-HPEA-EA) [9]. Oleuropein is the most abundant secoiridoid, representing up to 14% of olive fruit’s dry weight [7,10,11], followed by ligstroside, which is largely investigated for its role in chronic noncommunicable diseases [12]. Oleocanthal has been reported to induce selective antiproliferative and antimigrative effect on tumoral cell lines (MCF7 and MDA-MB-231) of different cancers [13]. Another class of olive oil phenols is represented by phenolic alcohols, mostly hydroxytyrosol (3,4-DHPEA) and tyrosol (p-HPEA) [14]. Tyrosol has been reported to exhibit long-term efficiency in inhibiting excessive production of reactive oxygen species (ROS), while hydroxytyrosol has displayed higher radical-scavenging activity [15]. Apigenin and luteolin are the main representatives of the flavonoids in olive oils, and low amounts of diosmetin, luteolin-7-glucoside, and rutin have also been reported [16,17]. These compounds can deactivate damaging free radicals, thus performing as potent antioxidants [18]. Another category of phenolic compounds is lignans. These compounds have been shown to be widely present within the minor polar compound fraction of olive oil [19]. Further research has shown that olive oil phenolic compounds exert possible chemoprotective and anticancer in vitro/in vivo effects in different types of cancers, such as human breast cancer (cell line MCF-7) [20], colon cancer (cell line HT115) [21], prostate cancer (cell lines LNCaP and C4-2) [22], and melanoma in C57BL/6N mice models [23].

Olive possesses four lipophilic isomers of tocopherols, with α-tocopherol as the dominant form, accounting for approximately 90% of all tocopherols, followed by the β- and γ-forms. δ-isomers have also been detected, albeit in minor amounts, in olive oil [24,25]. Tocopherols are considered among the most important free radical-scavenging antioxidant compounds in foods and other biological tissues [26,27,28].

Olive fruits and oil also contain squalene, a unique element for its antioxidant, detoxifying, and immunomodulatory properties and, above all, its chemopreventive and anticancer activity [29,30,31,32,33]. It is highly represented in olive oil, while other fruit oils, such as palm and avocado, possess lower amounts [24,34,35]. It is an important intermediary in the production of sterols and a precursor in cholesterol biosynthesis [34,35].

Sterols make up a large part of the fruit and the unsaponifiable fraction of olive oils, and they are exclusive to vegetable oils, as in animals, the main form is represented by cholesterol. β-sitosterol, covering approximately 90% of the total sterol content of oil, is essential for its bioactive properties against certain types of cancers such as breast, colon, prostate, and lung [36,37,38,39,40].

Among factors influencing the bioactive compounds of olive oil, genotype (also referred as cultivar or variety) certainly plays a major role, followed by climate, agronomical conditions, edaphic factors, and technologies applied for oil extraction [7,35,41,42,43,44,45]. Since olive counts on a very rich varietal heritage, represented by over 1200 main and over 3000 minor cultivars [46,47,48,49], a complete bioactive compound characterization of each cultivar has yet to come [6,42]. Understanding the differences of olive varieties for the content of bioactive compounds, selecting specific olive genotypes for certain metabolites, increasing quality, highlighting typicality, and valorizing positive attributes of the products [50,51,52] are of extreme importance. To evaluate the potential of different cultivars for their content in bioactive compounds, many studies have focused on the direct analysis of fruits instead of oils. Fatty acid methyl esters [6,53], phenols [44,54,55], tocopherols [56,57,58], phytosterols, and squalene [42,59] have been analyzed in olive fruits.

The present study aimed to characterize the main compounds produced by the prodigious metabolic machine represented by olive fruits, which is able to synthesize products unique to this species with anticancer, anti-inflammatory, and antineurodegenerative capacities and sensorial and taste properties. Our work consisted of exhaustive evaluation of an extensive panel of olive cultivars according to their phenol, tocopherol, squalene, and sterol profiles, defining the relative contribution of each factor and the genotype by environment interaction.

## 2. Materials and Methods

### 2.1. Plant Material

Fruit samples were collected from olive cultivars available at the International Olive Germplasm Collection (IOGC) of Zagaria (Enna, Italy) in two consecutive years. The collection was at 767 m above sea level, sited at 37.514° N; 14.296° E (decimal degrees). Temperature and precipitation data were collected along the two years to test their effects on trait variation. Daily average maximum and average minimum temperatures, as well as thermal amplitude and precipitation, were calculated from hourly data at the collection site.

Plant material under study included 61 cultivars, with two plants for each variety for a total of 122 trees. Three biological replicas were collected from each cultivar for a total of 183 samples. These varieties represented 12 countries (Table S1), and their identity was previously verified through SSR marker analysis and by comparison with data from the main olive collections [46,47,48,49].

Two kilograms of fruits was harvested from each tree. The harvesting time was set at the start of November, when 70% of fruits were at a ripening index (RI) of 2.0 (purple spots in less than half of fruit skin) [60] to standardize the conditions. Fruits were randomly chosen around the canopy and promptly transferred to the laboratory.

Analyzed compounds included the phenols hydroxytyrosol (HTYR), tyrosol (TYR), demethyloleuropein (D_OLEU), oleouropein (OLEU), oleocanthal (OLEOC), verbascoside (VER), rutin (RUT), luteolin -7-glycoside (LUT_7G), luteolin (LUE), and apigenin-7-glycoside (API_7G); squalene (SQU); the sterols campesterol (CAMP), stigmasterol (ST_STE), β-sitosterol (β_STE), and β-sitostanol (β_STA); and the tocopherols α-tocopherol (α_TOC), β-tocopherol (β_TOC), and γ-tocopherol (γ_TOC). All data are expressed as mg kg^−1^ of fresh fruit pulp. Oil content on fruit fresh weight (OCFFW) was also measured.

### 2.2. Extraction and Analysis of Fruit Phenolic Compounds

Fruit phenolic compounds were extracted according to a previously developed protocols [55,61]. One gram of fruit pulp was cut from 20 olive fruits for each sample and kept at 4 °C for 24 h in DMSO (6 mL/g of fruit pulp). The extracts were filtered through a 0.45 μm nylon mesh. After filtration, 50 µL of syringic acid (2.16 mg/mL) as internal standard was added to 950 µL of sample extract and kept at −20 °C until HPLC analysis.

### 2.3. Analysis of Phenolic Compounds through Reverse-Phase HPLC

The HPLC analyses of phenolic extracts were conducted according to Selvaggini et al. [62], with a reversed-phase column using a HPLC-DAD Varian ProStar-Diode Array Detector 330, composed of a vacuum degasser, a quaternary pump, an autosampler, a thermostatic column compartment, and a diode array detector (DAD), along with a 250 mm × 4.6 mm column 5 µm Kinetex Phenyl Hexyl 100A (Phenomenex, Torrance, CA, USA) as described in Mousavi et al. [63].

### 2.4. Analysis of Tocopherols

Tocopherol composition was determined by modifying the HPLC procedure described in Tura et al. [64]. First, 0.15 g of olive pulp was dissolved in 5 mL of hexane, homogenized by ultraturax, and then shacked in an ultrasonic bath for 30 min. Finally, samples were centrifuged for 5 min at 1500× *g* and conserved at −20 °C until HPLC analyses. Samples were then analyzed using the HPLC-DAD 330 Varian; the column was a KINETEX EVO C18 100 A—250 mm × 4.6 mm. The calibration curve was obtained by injecting standard solutions of (±)-α-tocopherol-synthetic, ≥96% (Sigma-Aldrich, St. Louis, MO, USA) at different concentrations. The HPLC analysis was performed using a mobile phase composed of Eluent “A” water with phosphoric acid 0.2% (Carlo Erba); eluent “B” was acetonitrile (Carlo Erba), at a ratio A/B 10:90. The flow rate was 1.2 mL/min, the injection volume was 30 µL, and the time of analysis was set for 20 min. Detection and quantification were performed at 290 nm [63].

### 2.5. Sterol and Squalene Analyses

Around 200 mg of ground olive pulp was placed in a 10 mL propylene tube. Two hundred microliters of an internal standard solution (5α-cholestan-3β-ol, Sigma-Aldrich) in hexane was added. The analysis of the unsaponifiable fraction by GC was previously reported in [6,59,63].

### 2.6. Oil Content Measurement

Data of oil content in fresh fruit pulp of the 61 studied cultivars (Table S1) were retrieved from a previous publication [6].

### 2.7. Statistical Analysis

General linear model including multivariate test, as well as the test between subjects and effects, were performed in IBM SPSS Statistics version 26. Descriptive statistics, violin plots, and boxplots were made by GraphPad Prism version 9.0.0 (GraphPad Software, San Diego, CA, USA, www.graphpad.com, accessed on 13 February 2022) to show the variability of each trait for 61 cultivars in two crop seasons. Correlations among the groups of phenols, squalene and sterols, and tocopherols were analyzed for the whole dataset using Pearson’s correlations (at *p* ≤ 0.05; *p* ≤ 0.01; *p* ≤ 0.001). The same correlation analysis was performed for all 19 traits with the climate data. A multiple regression model on the most studied traits (total phenols, total tocopherols, total sterols, squalene, and oil content of fruit fresh weight) and a principal component analysis (PCA) on the 19 chemical variables were performed through the latter program. All the analyses were done on three biological (from two trees) and three technical replicas for each cultivar.

## 3. Results

### 3.1. Trait Variation in a Common Set of Cultivars in Two Consecutive Crop Seasons

**Figure 1:**
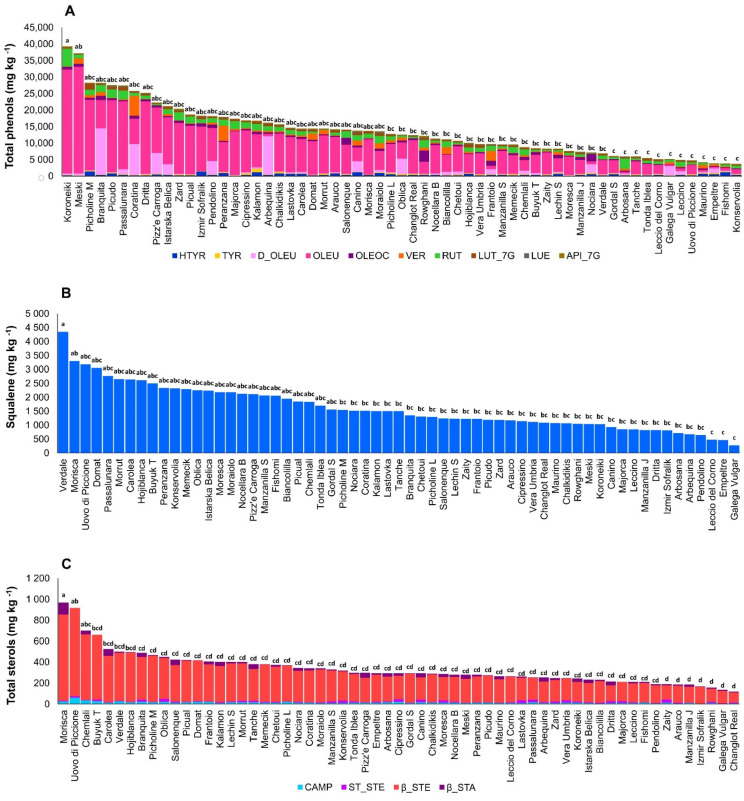

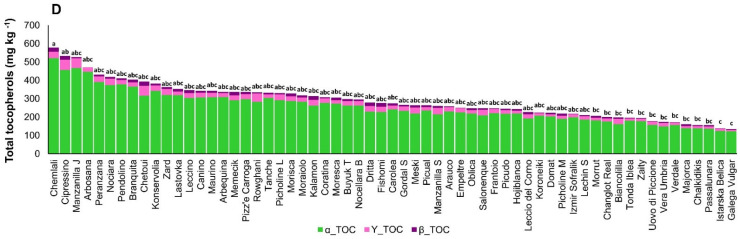
The variation in phenol (**A**), squalene (**B**), sterol (**C**), and tocopherol (**D**) content in 61 olive cultivars by two-year average (expressed as mg kg^−1^ of fresh fruit pulp). Different letters correspond to significantly different values of total phenols, total sterols, and total tocopherols at *p* ≤ 0.01.

**Figure 2:**
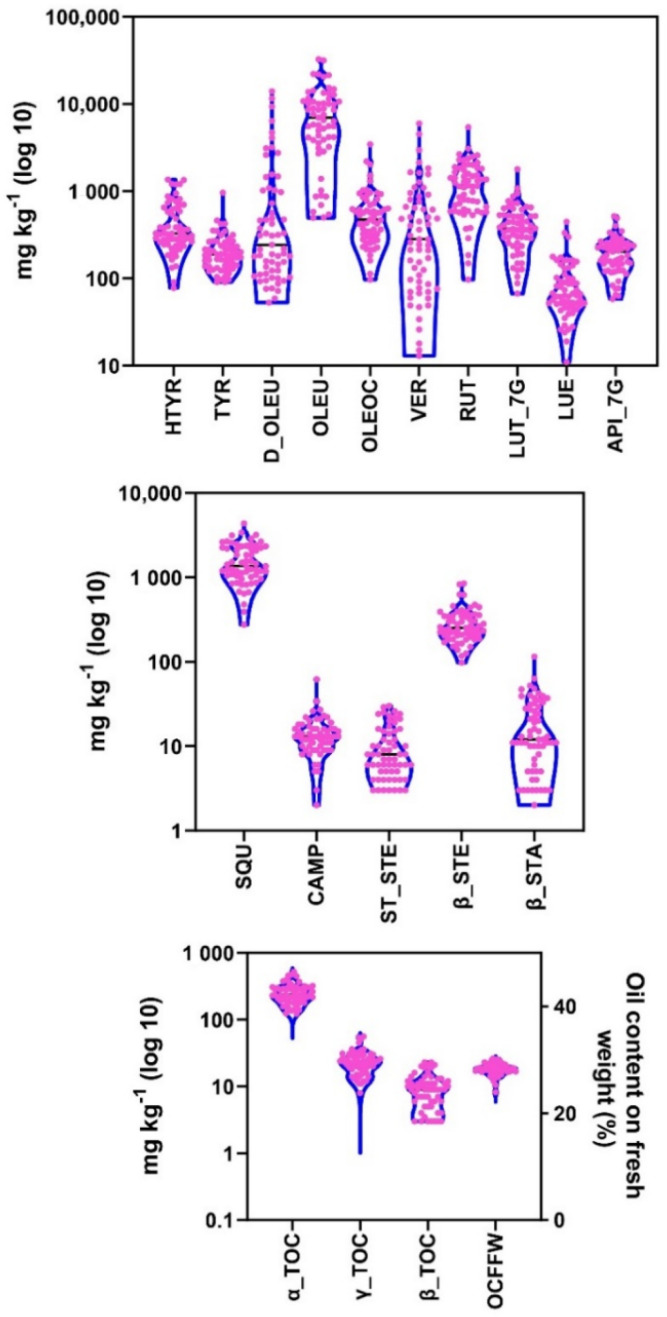
Violin graphs representing trait variation between two consecutive crop seasons. Each plot shows the distribution of data for 61 cultivars from the minimum to the maximum level, with horizontal inner line showing the data median. The horizontal width of the violin depends on the data density. The pink circles represent the 61 olive cultivars. The data are expressed as mg kg^−1^ of fruit pulp. The analyzed traits included phenols—hydroxytyrosol (HTYR), tyrosol (TYR), demethyloleuropein (D_OLEU), oleuropein (OLEU), oleocanthal (OLEOC), verbascoside (VER), rutin (RUT), luteolin -7-glycoside (LUT_7G), luteolin (LUE), apigenin-7-glycoside (API_7G); squalene and sterols—squalene (SQU), campesterol (CAMP), stigmasterol (ST_STE), β-sitosterol (β_STE), β-sitostanol (β_STA); tocopherols—α-tocopherol (α_TOC), γ-tocopherol (γ_TOC), β-tocopherol (β_TOC); and oil content on fruit fresh weight (OCFFW).

Analyses of all phenolic compounds in two crop seasons among 61 cultivars showed important variation in phenol content. The highest total phenol contents were detected in cvs. Dritta, Coratina, Passalunara, Picudo, Branquita, Picholine Marocaine, Meski, and Koroneiki, from 25,264 to 39,252 mg kg^−1^, while the cultivars with lowest total phenol content were Konservolia, Fishomi, and Empeltre, with content ranging from 3674 to 3831 mg kg^−1^ (Figure 1A). The main phenolic compound in all studied cultivars was OLEU, while in some cultivars, such as Branquita, Arbequina, Coratina, and Pizz’e Carroga, the main phenolic compound was D_OLEU (Figure 1A). The phenols with highest variation among 61 analyzed cultivars in two crop seasons were D_OLEU and VER, while the most stable ones were TYR and API_7G (Figure 2 and Table S2). In addition, variation in phenol content for the same cultivars in the two crop seasons was measured. The cultivars with highest variation between the two years of the experiment were Coratina, Picudo, Dritta, and Branquita. The most stable cultivars for their phenolic compounds in two years were Uovo di Piccione, Konservolia, Vera, Izmir Sofralik, Manzanilla de Jaen, and Empeltre (Table S2). Lignans, pinoresinol, and 1-acetoxypinoresinol were not detected in any cultivar in either of the two years.

Squalene analysis in all 61 cultivars and two consecutive years showed a significant difference between olive cultivars and crop seasons. The highest concentrations were found in cvs. Domat, Passalunara, Morisca and Verdale, with values ranging from 3132 to 4351 mg kg^−1^, and the cultivars with lowest quantity of this compost were Galega Vulgar, Empeltre, and Leccio del Corno, with values ranging from 274 to 478 mg kg^−1^ (Figure 1B). Among the cultivars, the highest variation in squalene content between the two crop seasons was measured in Buyuk Topakislak, Hojiblanca, and Passalunara, and the least variation was measured in Verdale, Arauco, and Izmir Sofralik (Table S3). Analysis of sterols showed, as with the previous bioactive compounds, considerable variation. The cultivars with highest content of total sterols were Buyuk Topakislak, Chemlali, Uovo di Piccione, and Morisca, with values ranging from 663 to 969 mg kg^−1^, while those with the lowest content were Changlot Real, Galega Vulgar, Rowghani, Izmir Sofralik, Manzanilla de Jaen, and Arauco, with values ranging from 119 to 186 mg kg^−1^ (Figure 1B). Among the analyzed sterols, β_STA had the highest variation when all 61 cultivars and both years of the experiment were considered (Figure 2). The cultivars with the least stability between the two crop seasons for this trait were Koroneiki, Meski, Tanche, and Kalamon, while more stable cultivars included Zaity, Arauco, Domat, Hojiblanca, Pendolino, and Konservolia (Table S3).

Total tocopherol content had less variation among the analyzed olive cultivars and the two crop seasons (Figure 1C). The highest quantities, in Arbosana, Manzanilla de Jaen, Cipressino, and Chemlali ranged from 471 to 579 mg kg^−1^, while the lowest quantities, in Galega Vulgar, Istarska Belica, Passalunara, and Chalkidikis, ranged from 135 to 156 mg kg^−1^. Among the 61 cultivars, those with the highest variation in total tocopherols between the two crop seasons were Zard, Pendolino, and Chemlali, and the most stable cultivars were Majorca, Verdale, Galega Vulgar, Rowghani, Dritta, and Peranzana. β_TOC had the highest variation in all 61 cultivars (Figure 2 and Table S4).

The highest percentages of OCFFW were calculated in cvs. Changlot Real, Biancolilla, Chalkidikis and Izmir Sofralik, ranging from 24.86 to 26.09%, while the lowest were calculated in cvs. Manzanilla de Jaen, Pendolino, Galega Vulgar, Empeltre, Meski, and Hojiblanca, ranging from 8.19 to 13.71% (Table S4). The cultivars with highest variation between the two crop seasons for this trait were Lastovka, Changlot Real, Konservolia, Koroneiki, and Buyuk Topakislak, and the most stable were Biancolilla, Carolea, Leccio del Corno, Morisca, Vera, Majorca, and Dritta (Table S4).

### 3.2. The Effect of Environment on the Trait Variation

**Figure 3:**
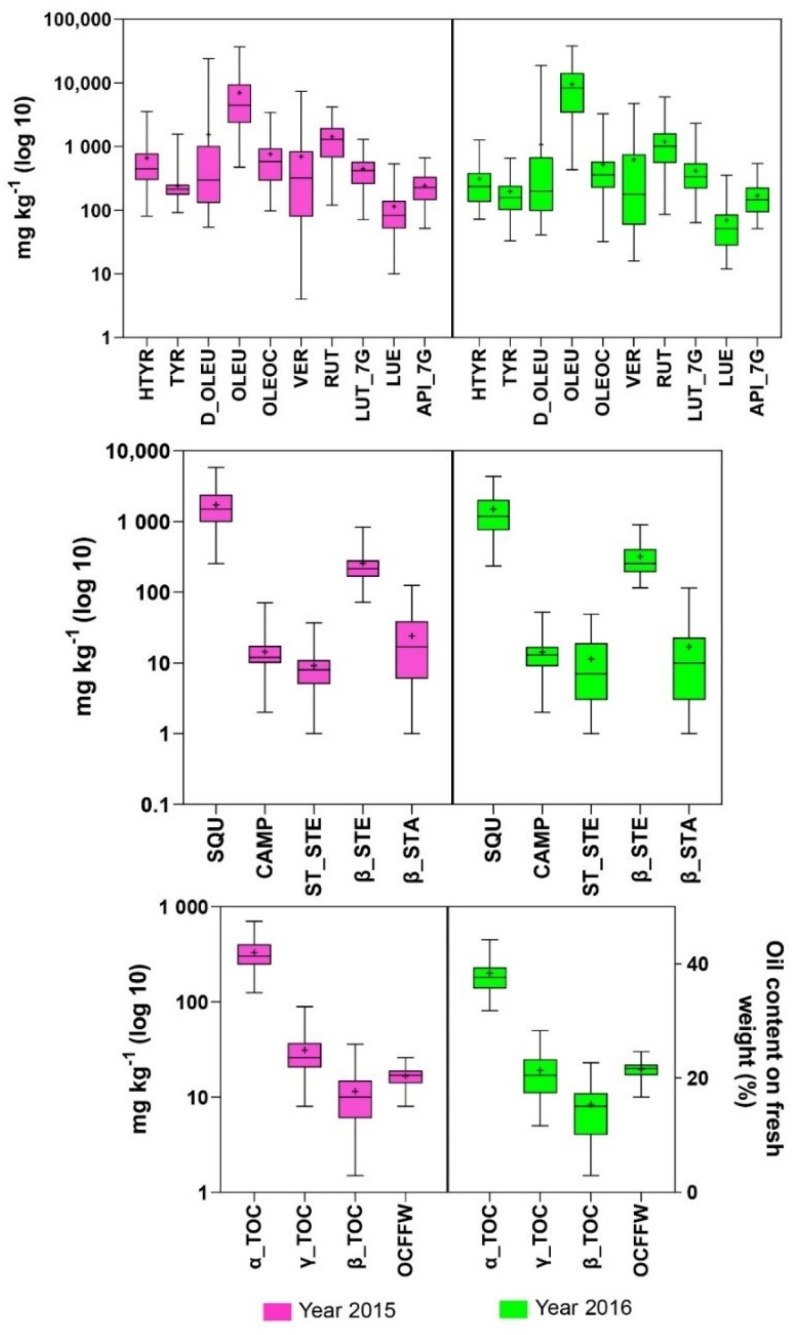
Box and whisker plots for trait variation in each crop season. Each plot shows the distribution of data for 61 cultivars from the minimum to the maximum level, with horizontal inner line showing the data median. The dot sign inside each box shows the mean value. The data are expressed as mg kg^−1^ of fruit pulp.

To study the effect of environment, metabolites were analyzed separately for each crop season in all 61 cultivars. Phenolic compounds showed high variability in both crop seasons (Figure 3; Tables S2 and S5). The highest variability in the two crop seasons was related to D_OLEU, OLEU, and VER. The variation of D_OLEU in the first year was 452-fold, and that in the second year was 667-fold, among the analyzed cultivars. OLEU had lower variation than D_OLEU in both years (110- and 103-fold in the first and second years, respectively). The maximum variation among these three traits was related to VER in the first year; VER had 2017-fold variability in the first year and 542-fold in the second year (Table S5). The sums of the phenolic compounds reflected lowest quantities of cvs. Konservolia, Fishomi, and Empeltre in the first year (from 3715 to 3724 mg kg^−1^) and cvs. Leccino, Konservolia, and Empeltre in the second year (from 3028 to 3912 mg kg^−1^). The maximum quantities of phenols were those of cvs. Picudo, Koroneiki, and Branquita in the first year (from 40,159 to 46,751 mg kg^−1^) and those of cvs. Dritta, Koroneiki, and Meski in the second year (from 35,058 to 43,144 mg kg^−1^) (Table S2).

The variation in squalene and sterols, in general, was lower than that in phenols in all studied cultivars and both crop seasons (Figure 3; Tables S3 and S5). Among them, the main variation was related to SQU and β_STE. The SQU variation among 61 cultivars was 10-fold in the first year and 19-fold in the second. The cultivars with the lowest amounts of SQU in the first year were Galega Vulgar and Leccio del Corno (253 and 503 mg kg^−1^, respectively), and in the second year, cv. Galega Vulgar again showed a low amount of this compound (296 mg kg^−1^). Contrariwise, the cultivars with maximum quantities of SQU in the first year were Verdale and Passalunara (4350 and 5834 mg kg^−1^, respectively), and cv. Verdale also had one of the highest quantities in the second year (4352 mg kg^−1^). The variation of β_STE was nine- and eightfold in the first and the second years among all the studied cultivars. The highest quantities of total sterols were measured in cvs. Uovo di Piccione and Morisca in both the first and second years (Figure 3 and Tables S3 and S5).

Among the analyzed tocopherols, the main variation was measured in γ_TOC and α_TOC (Figure 3 and Tables S4 and S5). γ_TOC variation among all analyzed cultivars in the first year was limited at 8-fold, while in the second year, it reached 66-fold. α_TOC had lower variation than γ_TOC at five- and ninefold in the first and second year, respectively. The total tocopherols were lowest in four olive varieties: cvs. Galega-Vulgar and Vera in the first year and cvs. Istarska Belica and Gordal Sevillana in the second year. The highest amounts were not detected in same cultivars between the two years of the experiment either. Cultivars Zard, Cipressino, and Chemlali had the highest total tocopherols in the first year, and cvs. Peranzana, Konservolia, and Manzanilla de Jaen had the highest in the second (Tables S4 and S5).

OCFFW was a stable trait between the two studied years (Figure 3 and Tables S4 and S5). The lowest percentages of OCFFW were in cvs. Manzanilla de Jaen and Pendolino in both crop seasons. The cultivars with maximum quantities of OCFFW were different between the two years of the experiment: Vera, Biancolilla, and Izmir Sofralik (from 24.12 to 25.74%) in the first year and Pizz’e Carroga, Chalkidikis, and Changlot Real (from 27.76 to 29.77%) in the second year.

### 3.3. Climate Effect on Trait Variation

**Figure 4:**
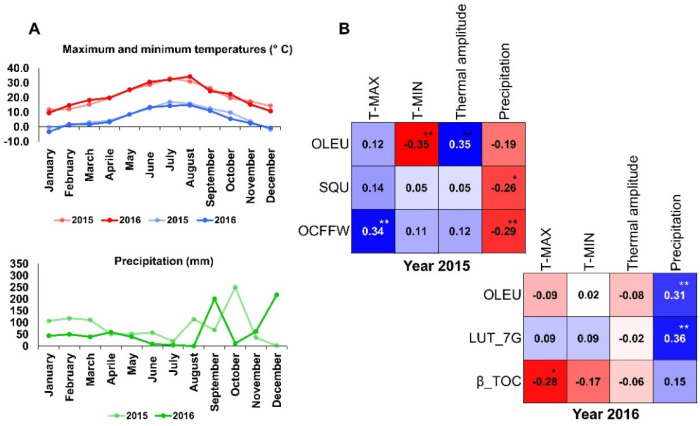
The monthly average temperatures and precipitation in two crop seasons (**A**). Red indicates maximum temperatures, and blue, minimum temperatures. Pearson’s correlation coefficients (**B**) between the hourly climate data and significantly correlated traits. Dark red and blue colors indicate significant data. Asterisks indicate ** p < 0.01 and * p < 0.05.

The maximum and minimum average monthly temperature, as well as precipitation, are shown in Figure 4A. Temperature data did not have significant differences between the studied years, while rainfall was 987.80 mm in 2015 and dropped to 746.40 mm in 2016. The correlations between climate data and all 19 traits were measured to individuate the most effective climate factor on trait variation (Figure 4B). In general, the *r* value was lower than 0.4 for all analyzed traits, and only a few compounds had significant positive or negative correlations with climate factors. In the first year of the study, OLEU had a negative correlation with minimum temperature and a positive correlation with the thermal amplitude; the same phenol had a positive correlation with precipitation in the second year. OCFFW had a positive correlation with maximum temperature in 2015 and was not correlated to this climate factor in 2016. The other phenol, LUT-7G, had a positive correlation with precipitation in 2016, the same as OLEU (Figure 4B).

### 3.4. The Effects of Genotype, Environment, and Their Interaction on Trait Variation

In order to identify the factors that influenced the studied traits’ variation, a multivariate test with all 61 cultivars and 19 traits in two consecutive years was performed. Genotype, environment, and their interaction had significant effects on trait variability, except for campesterol (CAMP), which reflected no significant effect of environment (Table 1 and Table S6). According to eta-squared (*η2*) values, genotypes with mean *η2* = 24.98% had the main effect on all 19 evaluated traits, with the highest effect on VER and D_OLEU and the lowest effect on OCFFW and α_TOC (Table S6). 

In fact, VER, with a minimum quantity of 13 mg kg^−1^ in cv. Konservolia and a maximum quantity of 5988 mg kg^−1^ in cv. Coratina, had more than 400-fold variation (Table S2). D_OLEU had the minimum quantity in cv. Moresca at 53 mg kg^−1^ and the highest content in cv. Branquita at 13,957 mg kg^−1^, with more than 250-fold variation. The environment had a major effect on HTYR, with twofold variation between the two studied years. G × E had an important effect on D_OLEU, HTYR, and ST_STE, even if this effect was lower than that of genotype (Table S6).

### 3.5. The Correlations among Traits and Their Linkage Analysis

The Pearson correlation coefficient was calculated for three different categories, phenols, squalene and sterols, and tocopherols (Figure S1). Significant and positive correlations were identified among the traits of each category. The phenolic compounds with the highest positive correlations were OLEU with rutin (RUT), *r = 0.47* (*p* ≤ 0.001), and LUE with API_7G, *r = 0.48* (*p* ≤ 0.001). In the SQU and sterol category, significant and positive correlations were found between CAMP and β_STE, *r = 0.80* (*p* < 0.001), and SQU and β_STE, *r = 0.52* (*p* ≤ 0.001). In the tocopherol category the most significant and positive correlation was between α_TOC and γ_TOC, *r = 0.67* (*p* ≤ 0.001) (Figure S1).

**Figure 5:**
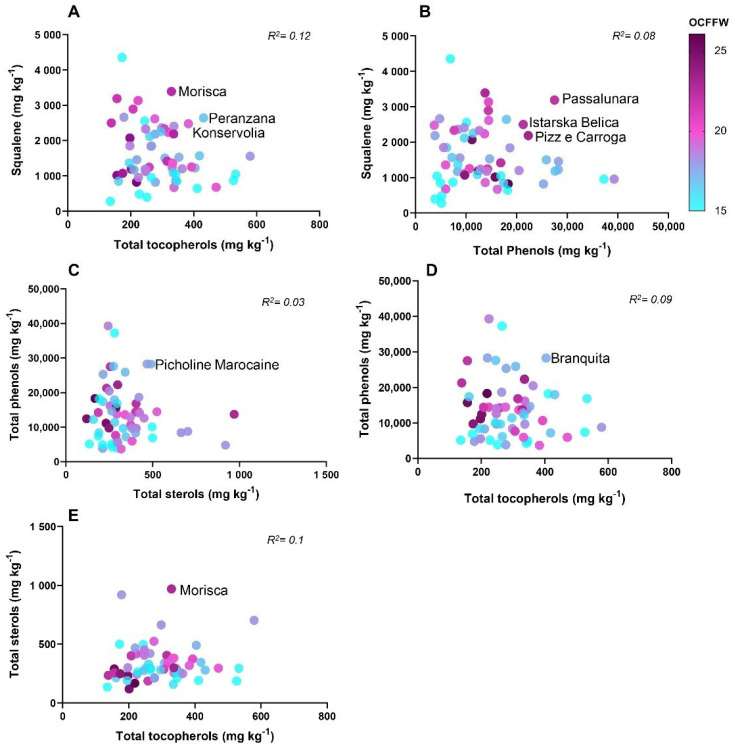
Scatter plot showing multiple regressions among the main traits (total phenols, total tocopherols, total sterols, squalene, and OCFFW). Data are expressed as mg kg^−1^ of fruit pulp. Violet circles indicate high content of OCFFW, and light blue circles indicate low content of OCFFW.

The main traits, total phenols, squalene, total sterols, total tocopherols, and OCFFW, were used to study the relationships among them and to identify promising genotypes regarding these traits. The multiple regression analysis for all combinations of traits, in general, showed a low value of *R^2^,* which evidenced the scarcity of cultivars with high values of combined traits, highlighting the need to obtain them through breeding. The multiple regression of squalene, total tocopherols, and OCFFW showed a low value of *R^2^ = 0.12* for all 61 cultivars. The cultivars with the highest values for these three traits were Morisca, Peranzana, and Konservolia (Figure 5A). The squalene, total phenol, and OCFFW combination had *R^2^ = 0.08*, and the cultivars with the highest values for this combination were Passalunara, Istarska Belica, and Pizz’e Carroga (Figure 5B). The most valuable cultivar for the combination of total phenols, total sterols, and OCFFW was Picholine Marocaine (*R^2^ = 0.03*) (Figure 5C). The total phenol, total tocopherol, and OCFFW combination had *R^2^ = 0.09*, and the most valuable cultivar for this combination was Branquita (Figure 5D). Finally, the cultivar Morisca demonstrated the highest value for the combination of total sterols, total tocopherols, and OCFFW, with *R^2^ = 0.1* (Figure 5E).

### 3.6. Clustering Olive Cultivars Based on the Studied Compounds

**Figure 6:**
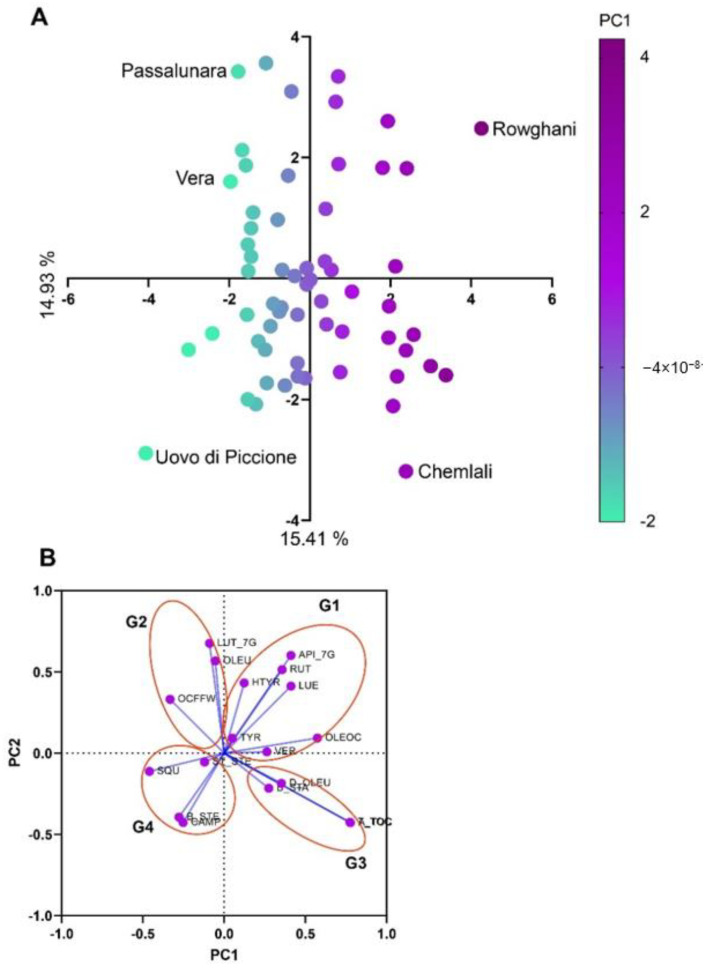
PCA (**A**) and loading plot (**B**) representing the distribution of the cultivars based on the 19 traits in two consecutive crop seasons. G1, cultivars rich in seven analyzed phenols and low in sterols; G2, cultivars rich in two phenolic compounds and OCFFW; G3, cultivars rich in tocopherols, one phenolic compound, and one sterol; and G4, cultivars rich in squalene and sterols.

Principal component analysis (PCA) was performed to cluster the 61 studied cultivars in two crop seasons for 19 analyzed traits (Figure 6A). The loading plot showed that the cultivars plotted in the group 1 (G1) area had the highest content of seven phenolic compounds out of eleven and low content of all other studied traits. Within this group, cv. Rowghani had the highest content for the seven analyzed phenols and the lowest content of sterols. The cultivars included in the second group (G2) had high content of OLEU and LUT_7G and high OCFFW. The G3 cultivars had high content of tocopherols, D_OLEU, and β_STA, and among them, cv. Chemlali had the highest quantity of all of these compounds. The G4 cultivars had high content of squalene and sterols, low content of phenols and tocopherols, and low oil content in fresh fruit. Cv. Uovo di Piccione had the highest quantity of squalene and sterols (Figure 6B).

## 4. Discussion

Cultivated olives show a remarkable genetic diversity among cultivars, and it has been proven how this variability is expressed in terms of biochemical composition of fatty acids [6], squalene and sterols [35,65], and phenols [7]. Most studies to date have focused on olive oil, because of the interest on the composition of the final product, but many other factors may affect the final balance of compounds in the oil, such as the enzymatic transformation from precursors that occurs during the extraction process, the length of fruit storage before extraction, the extraction method, the temperatures throughout the extraction process, the oil storage condition, and the packaging [7,63,66,67].

However, there is little knowledge on the content of secondary metabolites in the fruit, and even less on the differences among varieties. Chromatography techniques have revealed a high correlation between the compositions of olive fruit and olive oil in terms of fatty acids [42,53], phenols, tocopherols, sterols, and squalene [8,44].

Bioactive compounds can be affected by several factors. In the present study, we took under consideration the genotype and the main climatic parameters as main players controlling the metabolic profile of olive fruits.

In our study, growing site, agronomic management, and ripening index were the same for all cultivars. Rainfall and temperatures were analyzed in two crop seasons, but no strong correlation with these climate data was found. As in previous reports that showed negative correlations between water availability and phenol content [68,69], we found that OLEU and LUT_7G increased in the crop season with less precipitation.

The cultivar was the factor heavily impacting the concentration of most bioactive compounds, in accordance with previous studies that analyzed the variation of phenols [7,41,70], squalene and sterols [35,59], tocopherols [43,45] and OCFFW [6] and highlighted the high genotype effect.

Phenolic compounds were highly variable among all studied cultivars in both years of the experiment. In agreement with other studies [7,44,71], secoiridoid derivatives were the most abundant phenols in all evaluated cultivars. Secoiridoid derivatives are aglycon forms of secoiridoid glucosides formed during oil extraction by β-glucosidase enzymatic hydrolysis of oleuropein, demethyloleuropein and ligstroside [72,73]. The study of a large set of cultivars made it possible to pinpoint specific cultivars that possess potent radical scavenger and antioxidant properties, due to their high content in secoiridoids and flavonoids [70,71]. The cultivars with high content of the antioxidants, oleacein and oleuropein aglycon, and the anticancer and antiinflammatory potential, oleocanthal, were also identified [74,75]. It was interesting to note that some cultivars, which showed low-medium content of phenols were more stable for their phenolic compounds during the two years of observation. Contrariwise, the cultivars with high phenol content had the highest variation for this trait in two consecutive crop seasons.

The most represented phenols in fruit pulp were confirmed to be OLEU, D_OLEU, and RUT. Lignans, pinoresinol, and 1-acetoxypinoresinol, were not detected, in accordance with previous reports on olive fruit phenol profiling [44] in which the same method of phenol extraction (by DMSO) was applied. Furthermore, in a recent study published by the present authors [63], both lignans were detected in olive oil by the same instruments but via different methods of extraction. Among the studied cultivars, the Italian Coratina, Peranzana, Canino and Frantoio, with medium–high phenol content, showed very complex, rich phenolic profiles likely mirrored in oil derived from them [41,62,64]. The Spanish Branquita and Arbequina cultivars had the highest content of D_OLEU.

In the present study, 17 cultivars had more than 2000 mg kg^−1^ of squalene, and few had more than 3000 mg kg^−1^. This range of squalene confirmed what was reported in previous studies in olive oils and fruits [35,45,63,72,76,77]. The correlation with climate data did not show a strong effect on the variation of squalene among cultivars, though in the first year of the experiment, an increased quantity of this compound was correlated with the lower amount of precipitation. Squalene is essential for the biosynthesis of steroids and other triterpenes and, at the same time, constitutes an intermediate in the biosynthesis of phytosterols. Furthermore, much evidence has highlighted the numerous benefits of squalene to human health, such as anticancer, antioxidant, and cardioprotective activities [31,32,33,34].

The composition of the sterol fraction of olive oil is a very useful parameter for detecting adulteration or to check authenticity, since it can be considered as a fingerprint parameter [78,79,80]. In recent years, the sterol fraction of olive oil has received particular attention owing to its nutritional and health benefits, such as anticancer [37], antibacterial [38], and anti-inflammatory [39] effects. Our work showed large variation (9.4-fold) in sterol content among all cultivars. This variability could be explained only by the genetic component, since the variation between the two studied seasons was negligible. The strong genotype effect, as the main factor of controlling the sterol content, was also reported in a study on the sterol profiling of 43 olive cultivars [65] Cultivars with more than 500 mg kg^−1^ of total sterols (as Morisca, Uovo di Piccione, Chemlali and Buyuk Topakislak) could be of particular interest not only for olive oil but also for the production of table olives. In fact, the total amount of sterols corresponded to the highest content of β_STE, one of the most studied compounds for its beneficial effects on health [65,81,82].

The total content of tocopherols (135–579 mg kg^−1^), as well as those of different isoforms, was in agreement with the literature values [64,70,83]. The genotype effect on tocopherols was reported also in previous studies [26,64], even if α_TOC was also reported to be under environmental control [45,70]. The Tunisian cultivar Chemlali, with a high sterol content, also showed the highest level of total tocopherols (579 mg kg^−1^). The cultivars with very high values of total tocopherols are strong candidates from which to obtain olive oils with health-promoting activities, since vitamin E, as an antioxidant agent, limits lipid peroxidation in cell membranes and scavenges reactive oxygen species, protecting tissues from consequent oxidative damage [28]. Beneficial effects of supranutritional doses of vitamin E have been reported for cardiovascular diseases, cancer, chronic inflammation, and Alzheimer’s and Parkinson’s diseases [84,85].

Genotype had the highest effect on oil content in fruit fresh weight (OCFFW), while the effect of environment and its interaction was low and almost at the same level for *η2*. The correlation analysis with climate data did not show a strong effect of climate on oil content. A similar genotypic effect for oil content has been previously reported for olives in numerous cultivar trials [86,87,88]. A large variation among cultivars (8.16–26.09%) was observed. Six local and less diffused cultivars had values higher than 23%, and interestingly, two of them, cvs. Biancolilla and Vera, were among the cultivars with stable behavior for this trait.

Squalene, sterols, and tocopherols showed a high and positive intercorrelation, likely as a result of the existing interrelationships between components of the same biosynthetic pathway. They have been reported previously in olive [89] and in other oil crops [90]. The positive and significant correlation between β-sitosterol and campesterol is due to their common synthesis pathway [89], with a bifurcation that leads to the formation of β-sitosterol or campesterol. Previous research has shown that the orientation of the sterol biosynthetic flux towards sitosterol or campesterol is controlled mainly by the activity of two branch-point enzymes, SAM-24-methylene-lophenol-C-24-methyltransferase2 (SMT2) and C-4α-sterol-methyl-oxidase2 (SMO2), in plants [89]. Knowledge on the quantities of these sterols in the fruits of many olive cultivars may help to drive the choice of high-sterol cultivars.

The information on G × E interaction for metabolite content, widely studied in plants, should encourage the production of new cultivars with stable and superior phenotypes [7,91,92,93,94]. In fact, an important goal of crop improvement should be to minimize unfavorable G × E interaction and allow advantageous dynamic trait responses [6,95].

The climatic condition of the site (center Sicily) where sampled olive trees were grown, defined as “subtropical dry forest”, is common to vast areas of the Mediterranean [96,97], so the results obtained in this study take on a broader value and could be applicable in most olive-growing countries. Instead, in the near future (2021–2071), the climatic conditions of our experimental field could extend towards more northerly latitudes, which could become favorable for the establishment of olive cultivation to obtain fruits with the same metabolic values. The use of cultivars with high stability and concentration in bioactive compounds can decrease fluctuations in olive fruit and olive oil metabolic profiles. Indirect selection, based on the linkage between different traits, has simplified and accelerated the screening process [6,91,98]. The linkage among the most important olive metabolites and the scarcity of cultivars having high content of at least three traits would help to drive breeding programs toward the selection of the best and most stable cultivars for traits of interest.

## 5. Conclusions

Olive fruits contain very high quantities of bioactive compounds. Therefore, the in-depth study of the variability and amounts of these compounds in different cultivars is an important step to define the varietal allocation of these compounds. This study confirmed the significant genotype effect on all of the analyzed compounds, and it represents the first concerted effort to gather information on the most important bioactive metabolites in a large and representative sample of olive germplasm.

The need was also clearly evidenced to explore and characterize, at the metabolic level, local cultivars with peculiar bioactive compounds able to buffer environmental extremes due to climate and land-use changes. Among the olive varieties analyzed in this study, cv. Morisca, for the content of total tocopherols, squalene, total sterols, and OCFFW, as well as the varieties Koroneiki and Meski, for the quantity of total phenols, represent interesting sources of specific metabolites. The overall analysis confirmed the difficulty in finding cultivars with high values in a combination of bioactive compounds. Only a few, such as Chemlali, Rowghani, Passalunara, Vera, and Uovo di Piccione, were found.

The information derived from this work should contribute to selecting among different cultivars and breeding for new genotypes, focusing on specific metabolites with strong effects on human health.

## Figures and Tables

**Table 1:** Multivariate test from a general linear model for 61 olive cultivars in two crop seasons. The factors were significant, with values of *p* ≤ 0.000.

Effect	Value	F	Sig.
**Genotype**	309.107	453,357	0.000
**Environment**	57.62	212,280	0.000
**Genotype × Environment**	158.96	297,62	0.000

## Data Availability

Data is contained within the article and Supplementary Materials.

## Supplementary Materials

The following supporting information can be downloaded at: https://www.mdpi.com/article/10.3390/antiox11040672/s1, Figure S1: Pearson’s correlation coefficients among the phenolic compounds (A); squalene and sterols (B); and tocopherols (C) from all genotypes in two consecutive crop seasons. Violet indicates significant data, and light blue, nonsignificant data. Asterisks indicate *** *p* ≤ 0.000, ** *p* < 0.01, and * *p* < 0.05, Table S1: List of olive cultivars from IOGC, Table S2: Descriptive statistics of phenols (expressed as mg kg^−1^ of fresh fruit pulp) in 61 cultivars and two consecutive crop seasons, Table S3: Descriptive statistics of squalene and sterols (expressed as mg kg^−1^ of fresh fruit pulp) in 61 cultivars and two consecutive crop seasons, Table S4: Descriptive statistics of tocopherols (expressed as mg kg^−1^ of fresh fruit pulp) and oil content in fruit fresh weight (%) in 61 cultivars and two consecutive crop seasons, Table S5: Descriptive statistics by analysis of the nineteen chemical parameters (expressed as mg kg^−1^ of fresh fruit pulp) in 61 cultivars during two consecutive crop seasons, Table S6: Tests of between-subject effects from the general linear model for 61 olive cultivars in two consecutive crop seasons.
