# Supplementary material for: Bioactive Compound Profiling of Olive Fruit: The Contribution of Genotype
Source: Antioxidants (Basel). 2022 Mar 30;11(4):672. doi: 10.3390/antiox11040672 (PMC9032303; doi:10.3390/antiox11040672)
Supplement: Supplementary file 1 [file antioxidants-11-00672-s001.zip › antioxidants-1617945-supplementary.pdf]

A

	HTYR	TYR	D_OLEU	OLEU	OLEOC	VER	RUT	LUT_7G	LUE	API_7G
HTYR	1.00	0.35**	-0.13	0.01	0.04	0.07	-0.02	0.40***	0.26*	0.23
TYR	0.35**	1.00	-0.06	-0.01	0.10	-0.08	-0.05	0.06	-0.13	0.03
D_OLEU	-0.13	-0.06	1.00	-0.10	0.16	0.32**	0.01	-0.10	0.01	0.05
OLEU	0.01	-0.01	-0.10	1.00	0.02	-0.03	0.47***	0.36***	-0.06	0.27*
OLEOC	0.04	0.10	0.16	0.02	1.00	0.13	0.19	-0.07	0.26*	0.31**
VER	0.07	-0.08	0.32**	-0.03	0.13	1.00	0.04	-0.07	0.33**	-0.01
RUT	-0.02	-0.05	0.01	0.47***	0.19	0.04	1.00	0.35**	0.30**	0.42***
LUT_7G	0.40***	0.06	-0.10	0.36***	-0.07	-0.07	0.35**	1.00	0.24	0.40***
LUE	0.26*	-0.13	0.01	-0.06	0.26*	0.33**	0.30**	0.24	1.00	0.48***
API_7G	0.23	0.03	0.05	0.27*	0.31**	-0.01	0.42***	0.40***	0.48***	1.00

B

	SQU	CAMP	ST_STE	β_STE	β_STA
SQU	1.00	0.39**	0.06	0.52***	0.05
CAMP	0.39**	1.00	0.10	0.80***	0.05
ST_STE	0.06	0.10	1.00	-0.10	-0.02
β_STE	0.52***	0.80***	-0.10	1.00	0.27
β_STA	0.05	0.05	-0.02	0.27	1.00

C

	α_TOC	γ_TOC	β_TOC
α_TOC	1.00	0.67***	0.43***
γ_TOC	0.67***	1.00	0.49***
β_TOC	0.43***	0.49***	1.00

Figure S1. Pearson's correlation coefficients among the phenolic compounds (A); squalene and sterols (B); and tocopherols (C) from all genotypes in two consecutive crop seasons. The violet color indicates the significant data and the light blue color is for non-significant data. The asterisk indicates *** $p \leq 0.000$, ** $p < 0.01$, and * $p < 0.05$.

Table S1: List of olive cultivars from IOGC.

Cultivar	Country of origin
Arauco	Argentina
Arbequina	Spain
Arbosana	Spain
Biancolilla	Italy
Branquita	Spain
Bujuk Topakislak	Turkey
Canino	Italy
Carolea	Italy
Chalkidikis	Greece
Changlot Real	Spain
Chemlali	Tunisia
Chetoui	Tunisia
Cipressino	Italy
Coratina	Italy
Domat	Turkey
Dritta	Italy
Empeltre	Spain
Fishomi	Iran
Frantoio	Italy
Galega Vulgar	Portugal
Gordal Sevillana	Spain
Hojiblanca	Spain
Istarska Belica	Croatia
Izmir Sofralik	Turkey
Kalamata	Greece
Konservolia	Greece
Koroneiki	Greece
Lastovka	Croatia
Leccino	Italy
Leccio del Corno	Italy
Lechin de Sevilla	Spain
Majorca	Italy
Manzanilla de Jaen	Spain
Manzanilla de Sevilla	Spain
Maurino	Italy
Memecik	Turkey
Meski	Tunisia
Moraiolo	Italy
Moresca	Italy
Morisca	Spain
Morrut	Spain
Nocellara del Belice	Italy
Nociara	Italy
Oblica	Croatia

Passalunara	Italy
Pendolino	Italy
Peranzana	Italy
Picholine Languedoc	France
Picholine Marocaine	Morocco
Picual	Spain
Picudo	Spain
Pizz’e Carroga	Italy
Rowghani	Iran
Salonenque	France
Tanche	France
Tonda Iblea	Italy
Uovo di Piccione	Italy
Vera	Italy
Verdale	France
Zaity	Syria
Zard	Iran

Table S2. Descriptive statistics of phenols (expressed as mg kg^{-1} of fresh fruit pulp) in 61 cultivars and two consecutive crop seasons.

Cultivar	Descriptive statistics	HTYR	TYR	D OLEU	OLEU	OLEOC	VER	RUT	LUT 7G	LUE	API 7G	Total ph*	Total STD**
Arauco	Mean	820	160	136	10748	347	59	912	729	39	266	14,216	
	**STD**	**15**	**60**	**40**	**1456**	**10**	**27**	**210**	**92**	**1**	**55**		**447**
Arbequina	Mean	291	207	11556	692	528	448	1427	659	133	209	16,149	
	**STD**	**221**	**99**	**10022**	**147**	**139**	**242**	**213**	**148**	**160**	**70**		**3119**
Arbosana	Mean	224	97	490	593	323	603	3023	351	164	125	5,994	
	**STD**	**36**	**0**	**73**	**227**	**182**	**173**	**211**	**48**	**14**	**52**		**87**
Biancolilla	Mean	205	91	973	5224	215	2129	1742	375	99	83	11,135	
	**STD**	**22**	**9**	**71**	**668**	**22**	**32**	**77**	**38**	**1**	**3**		**203**
Branquita	Mean	320	185	13957	8577	1298	1093	2117	296	57	310	28,209	
	**STD**	**167**	**23**	**14146**	**11052**	**1223**	**195**	**411**	**171**	**31**	**16**		**5257**
Buyuk Topakislak	Mean	447	141	337	4933	1230	96	588	191	72	183	8,216	
	**STD**	**485**	**76**	**371**	**2508**	**1457**	**124**	**53**	**39**	**17**	**173**		**819**
Canino	Mean	1203	129	3179	4183	571	1564	1586	660	334	348	13,759	
	**STD**	**1388**	**56**	**901**	**442**	**274**	**869**	**330**	**79**	**91**	**200**		**446**
Carolea	Mean	638	142	460	9972	588	486	1312	457	163	223	14,442	
	**STD**	**196**	**79**	**3**	**10076**	**49**	**650**	**148**	**115**	**190**	**112**		**3137**
Chalkidikis	Mean	789	480	93	10112	367	299	1413	607	28	335	14,524	
	**STD**	**98**	**86**	**1**	**6202**	**84**	**52**	**571**	**317**	**2**	**213**		**1919**
Changlot Real	Mean	363	312	158	10037	562	78	379	318	52	150	12,409	
	**STD**	**66**	**124**	**166**	**8002**	**70**	**3**	**157**	**156**	**49**	**60**		**2501**
Chemlali	Mean	343	190	1092	3216	962	481	1843	395	24	202	8,747	
	**STD**	**21**	**109**	**126**	**800**	**19**	**40**	**211**	**112**	**10**	**81**		**236**
Chetoui	Mean	333	165	1080	7075	529	195	599	319	20	101	10,415	
	**STD**	**256**	**106**	**226**	**1970**	**76**	**179**	**35**	**87**	**5**	**33**		**594**
Cipressino	Mean	364	425	327	12720	466	213	1836	256	46	185	16,838	
	**STD**	**309**	**296**	**298**	**7584**	**71**	**280**	**1112**	**110**	**10**	**56**		**2330**
Coratina	Mean	193	165	9341	7615	960	5988	983	301	130	172	25,849	
	**STD**	**150**	**68**	**12443**	**4349**	**819**	**2002**	**433**	**142**	**45**	**18**		**3898**
Domat	Mean	345	121	65	8105	525	1518	636	493	145	279	12,233	
	**STD**	**340**	**65**	**26**	**8563**	**217**	**1331**	**56**	**81**	**24**	**2**		**2663**

Dritta	Mean	245	188	243	21992	613	18	1156	409	145	255	25,264	
	**STD**	**231**	**136**	**75**	**15221**	**219**	**15**	**605**	**228**	**148**	**180**		**4751**
Empeltre	Mean	526	325	1472	490	166	506	103	124	32	62	3,804	
	**STD**	**79**	**100**	**74**	**26**	**25**	**0**	**24**	**1**	**16**	**14**		**35**
Fishomi	Mean	1199	174	216	867	263	241	532	88	80	156	3,817	
	**STD**	**16**	**28**	**74**	**275**	**26**	**119**	**332**	**13**	**21**	**104**		**114**
Frantoio	Mean	397	215	1472	1018	1512	2955	1535	187	162	233	9,687	
	**STD**	**132**	**60**	**70**	**117**	**308**	**333**	**387**	**51**	**40**	**1**		**139**
Galega Vulgar	Mean	138	91	2755	501	113	620	691	131	11	58	5,109	
	**STD**	**22**	**1**	**212**	**23**	**16**	**81**	**38**	**11**	**2**	**8**		**65**
Gordal Sevillana	Mean	587	172	181	3851	322	15	592	236	52	119	6,126	
	**STD**	**636**	**76**	**92**	**67**	**61**	**4**	**452**	**149**	**57**	**74**		**207**
Hojiblanca	Mean	979	150	71	5088	263	346	1370	887	159	259	9,572	
	**STD**	**1012**	**54**	**23**	**2677**	**46**	**233**	**20**	**219**	**11**	**17**		**846**
Istarska belica	Mean	312	166	3096	14198	363	659	1487	640	26	320	21,268	
	**STD**	**196**	**12**	**2486**	**10734**	**113**	**4**	**136**	**332**	**3**	**158**		**3358**
Izmir Sofralik	Mean	1343	137	77	13431	930	171	1134	768	49	242	18,282	
	**STD**	**104**	**22**	**9**	**74**	**28**	**11**	**22**	**2**	**4**	**38**		**33**
Kalamon	Mean	1613	1114	633	10204	1033	592	2130	651	61	291	18,322	
	**STD**	**1720**	**647**	**693**	**828**	**1085**	**739**	**541**	**7**	**18**	**149**		**522**
Konservolia	Mean	390	195	93	1396	235	10	849	285	103	128	3,684	
	**STD**	**28**	**26**	**30**	**42**	**38**	**10**	**32**	**13**	**10**	**36**		**12**
Koroneiki	Mean	200	124	458	32777	828	82	5095	414	49	275	40,302	
	**STD**	**62**	**1**	**137**	**5626**	**76**	**45**	**1292**	**43**	**14**	**106**		**1760**
Lastovka	Mean	700	132	310	10628	571	259	1366	371	53	241	14,631	
	**STD**	**156**	**141**	**290**	**2980**	**590**	**139**	**788**	**42**	**10**	**265**		**891**
Leccino	Mean	425	145	1282	1005	447	296	1604	336	104	193	5,837	
	**STD**	**448**	**81**	**1104**	**487**	**587**	**354**	**532**	**180**	**58**	**141**		**315**
Leccio del Corno	Mean	293	267	123	2710	216	49	763	343	110	248	5,121	
	**STD**	**40**	**18**	**1**	**232**	**6**	**5**	**25**	**25**	**3**	**11**		**70**
Lechin de Sevilla	Mean	652	214	375	4130	1023	913	569	191	66	158	8,289	
	**STD**	**119**	**31**	**53**	**768**	**245**	**127**	**110**	**52**	**19**	**17**		**227**
Majorca	Mean	78	120	155	13208	213	505	2387	533	64	122	17,385	
	**STD**	**2**	**8**	**10**	**103**	**10**	**17**	**275**	**80**	**1**	**31**		**85**
Manzanilla de Jaen	Mean	316	236	195	4101	798	51	800	421	101	213	7,231	

	**STD**	**19**	**13**	**8**	**108**	**74**	**2**	**16**	**12**	**1**	**34**		**35**
Manzanilla de Sevilla	Mean	378	163	103	6957	422	281	775	295	93	166	9,634	
	**STD**	**322**	**32**	**38**	**490**	**273**	**273**	**445**	**3**	**7**	**85**		**186**
Maurino	Mean	693	268	308	581	421	1108	588	67	68	117	4,220	
	**STD**	**288**	**101**	**53**	**42**	**89**	**260**	**106**	**6**	**27**	**34**		**97**
Memecik	Mean	280	273	172	5693	622	61	1895	538	90	217	9,841	
	**STD**	**74**	**59**	**49**	**568**	**53**	**6**	**373**	**73**	**11**	**1**		**189**
Meski	Mean	98	127	536	29570	931	1533	1041	237	68	118	34,260	
	**STD**	**31**	**54**	**113**	**11761**	**51**	**422**	**261**	**5**	**25**	**20**		**3687**
Moraiolo	Mean	872	233	729	5770	691	1642	2275	619	445	335	13,613	
	**STD**	**955**	**10**	**188**	**5826**	**374**	**629**	**619**	**72**	**128**	**27**		**1765**
Moresca	Mean	133	97	53	5538	322	210	721	384	40	123	7,621	
	**STD**	**0**	**38**	**5**	**618**	**62**	**32**	**69**	**65**	**21**	**24**		**186**
Morisca	Mean	301	109	73	5837	239	116	1335	610	25	225	8,871	
	**STD**	**9**	**7**	**2**	**6833**	**23**	**11**	**15**	**30**	**1**	**7**		**2157**
Morrut	Mean	329	433	98	11631	382	1420	196	100	67	81	14,737	
	**STD**	**11**	**53**	**18**	**704**	**45**	**633**	**48**	**24**	**23**	**24**		**270**
Nocellara del Belice	Mean	288	197	316	8558	429	362	659	255	64	248	11,377	
	**STD**	**54**	**36**	**109**	**386**	**30**	**20**	**161**	**31**	**1**	**63**		**115**
Nociara	Mean	620	351	2605	867	2190	76	149	127	46	147	7,177	
	**STD**	**257**	**6**	**159**	**75**	**334**	**16**	**17**	**12**	**11**	**16**		**119**
Oblica	Mean	279	267	4708	4965	563	955	593	149	28	77	12,585	
	**STD**	**28**	**15**	**5621**	**3125**	**407**	**1225**	**2**	**12**	**14**	**36**		**1887**
Passalunara	Mean	385	148	1554	20535	366	281	2643	1077	178	285	27,451	
	**STD**	**443**	**88**	**1322**	**6739**	**209**	**311**	**110**	**186**	**217**	**182**		**2055**
Pendolino	Mean	282	222	4094	9932	1037	615	1415	345	54	186	18,182	
	**STD**	**224**	**25**	**3101**	**7604**	**84**	**569**	**412**	**67**	**2**	**130**		**2429**
Peranzana	Mean	946	174	193	8944	449	4523	1894	448	162	213	17,946	
	**STD**	**182**	**56**	**176**	**10994**	**325**	**307**	**175**	**132**	**170**	**13**		**3424**
Picholine Languedoc	Mean	582	355	221	8527	424	759	1146	527	52	228	12,822	
	**STD**	**144**	**144**	**177**	**1411**	**188**	**759**	**343**	**272**	**27**	**194**		**417**
Picholine Marocaine	Mean	1905	375	134	23529	652	677	1985	1527	114	209	31,107	
	**STD**	**2325**	**253**	**101**	**9219**	**157**	**521**	**1435**	**1117**	**135**	**205**		**2793**
Picual	Mean	325	180	122	14309	690	177	1843	374	89	200	18,308	
	**STD**	**292**	**71**	**112**	**1861**	**461**	**68**	**728**	**73**	**5**	**100**		**568**

Picudo	Mean	764	263	102	21817	599	132	2570	734	174	399	27,554	
	**STD**	**656**	**141**	**6**	**12881**	**583**	**118**	**2245**	**788**	**36**	**374**		**3954**
Pizz'e Carroga	Mean	485	179	6389	13717	626	70	369	149	53	227	22,264	
	**STD**	**207**	**93**	**8412**	**3254**	**284**	**26**	**79**	**57**	**44**	**88**		**2703**
Rowghani	Mean	407	201	150	3406	3351	829	2226	509	275	470	11,824	
	**STD**	**18**	**1**	**7**	**215**	**121**	**64**	**96**	**11**	**35**	**29**		**68**
Salonenque	Mean	300	239	200	8943	2044	24	2037	377	66	62	14,291	
	**STD**	**66**	**53**	**93**	**535**	**85**	**9**	**168**	**78**	**20**	**5**		**157**
Tanche	Mean	179	238	180	3885	275	86	537	357	43	147	5,927	
	**STD**	**67**	**71**	**26**	**478**	**8**	**0**	**145**	**9**	**27**	**47**		**144**
Tonda iblea	Mean	238	132	106	3126	230	70	801	521	87	226	5,538	
	**STD**	**193**	**74**	**50**	**594**	**81**	**46**	**510**	**344**	**70**	**204**		**201**
Uovo di Piccione	Mean	245	191	87	2856	97	97	571	441	52	123	4,758	
	**STD**	**13**	**6**	**2**	**13**	**2**	**2**	**23**	**23**	**3**	**1**		**9**
Vera	Mean	235	260	113	6335	300	68	1248	868	44	233	9,702	
	**STD**	**7**	**8**	**15**	**43**	**30**	**33**	**17**	**28**	**8**	**4**		**13**
Verdale	Mean	164	216	155	4331	185	34	1132	444	62	142	6,866	
	**STD**	**6**	**17**	**35**	**144**	**4**	**14**	**103**	**62**	**23**	**22**		**46**
Zaity	Mean	371	178	147	5406	331	265	299	197	64	155	7,414	
	**STD**	**390**	**103**	**101**	**4802**	**156**	**83**	**42**	**78**	**58**	**16**		**1486**
Zard	Mean	263	175	298	12524	919	51	2567	499	74	506	17,876	
	**STD**	**180**	**71**	**133**	**12019**	**849**	**17**	**399**	**73**	**2**	**53**		**3747**

* Total phenols represented the sum of all analyzed phenolic compounds for each cultivar.

** Total STD is a standard deviation of all phenols calculated from each STD.

Table S3. Descriptive statistics of squalene and sterols (expressed as mg kg^{-1} of fresh fruit pulp) in 61 cultivars and two consecutive crop seasons.

Cultivar	Descriptive statistics	SQU	CAMP	ST STE	β STE	β STA	Total ST*	Total STD**
Arauco	Mean	1241	6.26	5.79	164.41	9.93	186.39	
	**STD**	**8.85**	**0.03**	**4.50**	**0.98**	**0.10**		**3.80**
Arbequina	Mean	666	11.54	7.07	196.10	39.03	253.75	
	**STD**	**237.49**	**5.99**	**4.26**	**26.61**	**8.31**		**101.55**
Arbosana	Mean	717	17.02	9.31	236.57	36.25	299.15	
	**STD**	**180.31**	**4.39**	**3.48**	**4.52**	**15.20**		**77.70**
Biancolilla	Mean	2068	5.42	7.45	205.82	12.24	230.92	
	**STD**	**85.18**	**0.59**	**0.78**	**10.50**	**1.08**		**36.89**
Branquita	Mean	1222	22.05	19.20	406.91	42.04	490.20	
	**STD**	**46.49**	**6.74**	**2.87**	**72.67**	**5.01**		**31.39**
Buyuk Topakislak	Mean	1948	22.67	11.78	498.03	1.92	534.39	
	**STD**	**1740**	**20.05**	**11.80**	**513.96**	**1.21**		**749.42**
Canino	Mean	858	16.42	24.27	215.31	37.44	293.43	
	**STD**	**409.40**	**5.58**	**23.50**	**70.13**	**22.71**		**171.15**
Carolea	Mean	2613	13.19	5.75	441.60	63.38	523.92	
	**STD**	**264.63**	**4.88**	**1.83**	**283.22**	**16.50**		**146.05**
Chalkidikis	Mean	1087	15.88	4.65	272.51	7.48	300.51	
	**STD**	**325.61**	**4.44**	**3.04**	**40.27**	**1.38**		**141.06**
Changlot Real	Mean	1191	5.33	7.55	98.44	8.17	119.48	
	**STD**	**231.93**	**0.55**	**8.34**	**37.79**	**1.88**		**99.45**
Chemlali	Mean	1553	34.45	4.42	626.58	36.77	702.22	
	**STD**	**407.15**	**16.36**	**4.35**	**266.96**	**2.03**		**187.22**
Chetoui	Mean	1293	12.80	7.79	295.35	20.26	336.20	
	**STD**	**187.10**	**3.66**	**6.54**	**177.57**	**20.25**		**94.58**
Cipressino	Mean	1046	21.69	24.68	224.19	23.77	294.33	
	**STD**	**710.45**	**7.63**	**21.17**	**44.66**	**27.49**		**306.72**
Coratina	Mean	1499	13.23	3.66	304.40	19.38	340.66	
	**STD**	**816.19**	**6.59**	**1.64**	**68.94**	**3.00**		**357.17**
Domat	Mean	3052	18.27	5.99	340.66	1.57	366.49	
	**STD**	**336.83**	**10.11**	**0.80**	**211.20**	**0.20**		**154.63**
Dritta	Mean	817	11.38	16.38	151.34	35.32	214.42	
	**STD**	**629.02**	**0.49**	**8.77**	**8.95**	**17.54**		**277.38**
Empeltre	Mean	468	14.90	9.51	285.91	21.71	332.03	
	**STD**	**328.50**	**8.88**	**1.62**	**117.42**	**25.45**		**137.83**
Fishomi	Mean	2181	14.95	2.69	183.39	9.61	210.64	
	**STD**	**1157**	**0.65**	**1.14**	**25.15**	**8.32**		**513.74**
Frantoio	Mean	1119	19.18	5.88	353.85	25.72	404.63	
	**STD**	**519.60**	**6.42**	**3.68**	**32.09**	**27.83**		**224.90**
Galega Vulgar	Mean	274	2.59	4.50	116.81	11.45	135.35	
	**STD**	**30.52**	**0.12**	**0.71**	**1.69**	**0.69**		**13.30**
Gordal Sevillana	Mean	1843	9.49	3.82	278.30	2.26	293.88	
	**STD**	**50.04**	**0.12**	**4.26**	**102.57**	**0.93**		**44.88**
Hojiblanca	Mean	2060	14.04	2.28	389.48	2.17	407.97	
	**STD**	**2097**	**12.31**	**0.54**	**358.77**	**0.24**		**909.20**
Istarska belica	Mean	2497	8.57	8.91	184.42	32.28	234.17	
	**STD**	**232.71**	**3.54**	**5.51**	**34.45**	**7.41**		**99.18**
Izmir Sofralik	Mean	818	1.94	1.06	162.73	1.64	167.38	
	**STD**	**12.64**	**0.22**	**0.08**	**21.42**	**0.91**		**9.63**
Kalamon	Mean	1700	10.06	13.72	385.85	49.70	459.32	

	**STD**	**1213**	**7.77**	**15.86**	**167.13**	**43.41**		**520.11**
Konservolia	Mean	2296	10.86	21.29	251.60	5.45	289.19	
	**STD**	**764.85**	**2.40**	**1.22**	**120.34**	**0.45**		**332.16**
Koroneiki	Mean	928	12.39	11.32	186.22	23.87	233.79	
	**STD**	**103.39**	**0.21**	**0.37**	**9.56**	**31.40**		**43.49**
Lastovka	Mean	1525	8.58	26.27	217.60	11.41	263.86	
	**STD**	**356.89**	**7.51**	**15.82**	**86.17**	**2.94**		**150.88**
Leccino	Mean	1071	10.35	9.60	199.30	18.88	238.13	
	**STD**	**952.34**	**6.25**	**7.44**	**76.59**	**24.59**		**414.04**
Leccio del Corno	Mean	478	10.91	6.06	244.90	2.50	264.37	
	**STD**	**34.93**	**0.59**	**4.21**	**18.24**	**0.71**		**14.85**
Lechin de Sevilla	Mean	1168	16.36	9.58	362.77	12.78	401.49	
	**STD**	**460.85**	**3.12**	**1.31**	**129.20**	**13.96**		**197.01**
Majorca	Mean	846	8.42	19.51	182.70	2.50	213.13	
	**STD**	**19.76**	**0.38**	**4.86**	**6.02**	**0.71**		**7.90**
Manzanilla de Jaen	Mean	810	8.59	5.70	155.88	22.64	192.81	
	**STD**	**78.63**	**0.59**	**0.43**	**8.65**	**0.50**		**34.21**
Manzanilla de Sevilla	Mean	2116	9.01	13.77	297.67	9.91	330.36	
	**STD**	**1240**	**2.65**	**11.84**	**138.59**	**7.01**		**539.51**
Maurino	Mean	1039	10.12	3.31	223.73	27.42	264.59	
	**STD**	**581.31**	**2.15**	**1.70**	**40.69**	**23.54**		**252.88**
Memecik	Mean	2131	12.57	7.30	317.68	1.99	339.55	
	**STD**	**1158**	**3.32**	**2.45**	**170.04**	**0.70**		**503.38**
Meski	Mean	1145	11.62	2.40	239.79	48.66	302.48	
	**STD**	**825.14**	**6.82**	**0.14**	**52.87**	**35.78**		**358.97**
Moraiolo	Mean	2234	11.50	6.34	312.09	10.36	340.29	
	**STD**	**693.92**	**3.45**	**6.14**	**68.52**	**6.45**		**302.12**
Moresca	Mean	2333	13.89	21.09	221.77	29.44	286.19	
	**STD**	**830.90**	**0.43**	**4.27**	**3.49**	**21.23**		**368.39**
Morisca	Mean	3300	22.14	7.49	829.60	119.89	979.11	
	**STD**	**126.14**	**1.61**	**0.73**	**7.64**	**7.22**		**54.58**
Morrut	Mean	2769	15.30	15.20	306.93	15.24	352.67	
	**STD**	**514.09**	**2.59**	**6.29**	**209.79**	**13.59**		**221.99**
Nocellara del Belice	Mean	2251	12.50	10.00	237.50	20.50	280.50	
	**STD**	**419.31**	**0.71**	**1.41**	**23.33**	**0.71**		**184.85**
Nociara	Mean	1566	14.22	5.49	297.43	27.59	344.73	
	**STD**	**575.65**	**1.23**	**3.00**	**101.21**	**7.20**		**248.46**
Oblica	Mean	2325	20.57	29.55	388.94	10.70	449.76	
	**STD**	**1511**	**0.64**	**27.43**	**184.35**	**12.84**		**654.85**
Passalunara	Mean	3187	15.90	24.41	209.05	5.46	254.82	
	**STD**	**3744**	**3.97**	**25.59**	**111.80**	**4.90**		**1658.44**
Pendolino	Mean	641	6.35	3.68	170.06	11.17	191.27	
	**STD**	**489.52**	**3.25**	**2.38**	**34.50**	**0.24**		**214.87**
Peranzana	Mean	2639	12.71	3.81	247.09	15.47	279.08	
	**STD**	**133.70**	**3.19**	**0.44**	**81.10**	**7.42**		**59.83**
Picholine Languedoc	Mean	1234	19.62	4.40	342.76	4.07	370.85	
	**STD**	**810.35**	**12.16**	**0.24**	**155.28**	**0.85**		**349.74**
Picholine Marocaine	Mean	1506	17.90	2.23	421.22	6.98	448.32	
	**STD**	**218.02**	**0.55**	**0.38**	**85.10**	**6.40**		**94.26**
Picual	Mean	1519	15.65	3.09	336.62	5.50	360.86	
	**STD**	**1351**	**8.56**	**0.44**	**239.71**	**3.51**		**584.80**
Picudo	Mean	1182	12.01	5.97	251.51	4.72	274.21	
	**STD**	**280.05**	**0.06**	**4.20**	**41.08**	**4.55**		**120.81**
Pizz’e Carroga	Mean	2184	12.72	4.94	232.36	48.33	298.34	

	**STD**	**522.45**	**2.57**	**1.64**	**50.61**	**21.31**		**226.01**
Rowghani	Mean	1033	12.40	8.96	142.49	12.64	176.49	
	**STD**	**67.05**	**3.67**	**1.35**	**24.76**	**0.91**		**28.35**
Salonenque	Mean	1314	14.11	14.51	342.87	57.60	429.09	
	**STD**	**592.93**	**4.14**	**9.96**	**15.10**	**23.02**		**259.42**
Tanche	Mean	1355	15.99	14.52	303.79	47.16	381.45	
	**STD**	**756.99**	**11.87**	**4.87**	**259.19**	**39.84**		**320.86**
Tonda iblea	Mean	1846	15.00	8.18	264.40	11.05	298.63	
	**STD**	**287.57**	**2.75**	**2.13**	**5.74**	**11.67**		**126.17**
Uovo di Piccione	Mean	2659	61.86	12.36	843.28	1.37	918.87	
	**STD**	**153.21**	**13.26**	**3.31**	**79.71**	**0.89**		**66.10**
Vera	Mean	1068	12.03	22.77	208.77	4.30	247.87	
	**STD**	**107.65**	**0.46**	**2.65**	**22.24**	**3.02**		**45.82**
Verdale	Mean	4351	25.54	6.18	456.59	11.04	499.36	
	**STD**	**1.34**	**6.31**	**1.15**	**7.65**	**1.48**		**3.14**
Zaity	Mean	1498	12.40	22.82	144.13	11.00	190.36	
	**STD**	**573.88**	**1.26**	**25.81**	**28.00**	**0.00**		**250.84**
Zard	Mean	1222	8.62	12.46	185.23	20.59	226.90	
	**STD**	**104.76**	**0.24**	**13.40**	**80.81**	**1.33**		**49.10**

[*] Total sterols for each cultivar.

[**] Total STD is a standard deviation of squalene and sterols from each STD.

Table S4. Descriptive statistics of tocopherols (expressed as mg kg^{-1} of fresh fruit pulp) and oil content in fruit fresh weight (%) in 61 cultivars and two consecutive crop seasons.

Cultivar	Descriptive statistics	α_TOC	γ_TOC	β_TOC	OCFFW	Total TOC*	Total TOC STD**
Arauco	Mean	230.42	23.31	3.90	21.52	257.64	
	**STD**	**30.89**	**1.86**	**3.96**	**0.69**		**16.19**
Arbequina	Mean	308.66	22.38	5.83	19.30	336.87	
	**STD**	**179.93**	**6.46**	**8.24**	**3.92**		**99.64**
Arbosana	Mean	474.05	23.39	0.63	19.73	498.07	
	**STD**	**118.18**	**3.03**	**0.88**	**0.32**		**67.11**
Biancolilla	Mean	161.19	28.92	7.25	25.01	197.36	
	**STD**	**17.30**	**9.19**	**3.89**	**0.02**		**6.76**
Branquita	Mean	365.52	24.04	14.10	17.37	403.66	
	**STD**	**114.15**	**1.94**	**2.97**	**3.55**		**64.49**
Buyuk Topakislak	Mean	310.86	30.63	10.58	16.47	352.07	
	**STD**	**205.20**	**24.41**	**0.81**	**7.73**		**111.81**
Canino	Mean	306.91	25.11	11.15	19.46	343.16	
	**STD**	**42.63**	**4.11**	**6.08**	**1.77**		**21.69**
Carolea	Mean	241.43	21.42	12.88	19.54	275.73	
	**STD**	**43.39**	**2.78**	**9.73**	**0.12**		**21.72**
Chalkidikis	Mean	151.84	14.62	4.29	23.80	170.75	
	**STD**	**54.40**	**10.05**	**1.82**	**6.36**		**28.28**
Changlot Real	Mean	175.30	16.43	9.03	24.86	200.76	
	**STD**	**87.85**	**10.28**	**9.37**	**6.94**		**45.05**
Chemlali	Mean	520.88	34.89	23.37	18.16	579.14	
	**STD**	**254.15**	**9.88**	**0.17**	**4.61**		**143.92**
Chetoui	Mean	343.38	57.14	23.26	20.38	423.77	
	**STD**	**116.84**	**12.54**	**0.20**	**0.33**		**64.08**
Cipressino	Mean	455.86	56.44	21.12	15.26	533.42	
	**STD**	**236.35**	**45.53**	**7.85**	**3.32**		**122.51**
Coratina	Mean	276.52	25.52	6.00	17.05	308.04	
	**STD**	**143.97**	**20.73**	**6.16**	**2.69**		**75.71**
Domat	Mean	242.35	13.09	9.10	20.05	264.53	
	**STD**	**166.80**	**6.98**	**1.27**	**1.53**		**93.96**
Dritta	Mean	229.89	29.51	18.82	17.55	278.22	
	**STD**	**7.44**	**7.08**	**2.35**	**0.18**		**2.84**
Empeltre	Mean	238.50	27.51	2.85	13.21	268.87	
	**STD**	**55.57**	**20.24**	**0.92**	**1.79**		**27.72**
Fishomi	Mean	227.58	29.03	20.19	17.69	276.79	
	**STD**	**83.08**	**29.44**	**22.76**	**0.99**		**33.07**
Frantoio	Mean	221.42	21.94	3.40	18.94	246.76	
	**STD**	**97.61**	**0.31**	**2.26**	**0.63**		**55.62**
Galega Vulgar	Mean	122.46	8.08	4.11	12.09	134.65	
	**STD**	**2.89**	**0.28**	**0.13**	**1.34**		**1.55**
Gordal Sevillana	Mean	233.88	23.15	9.12	16.33	266.16	
	**STD**	**196.87**	**16.58**	**4.35**	**5.03**		**107.79**
Hojiblanca	Mean	242.19	16.07	11.74	13.04	270.00	
	**STD**	**96.93**	**5.28**	**7.44**	**2.81**		**52.30**
Istarska belica	Mean	124.40	11.09	2.73	22.37	138.21	
	**STD**	**61.04**	**8.39**	**2.16**	**4.95**		**32.34**
Izmir Sofralik	Mean	198.80	15.69	3.30	26.09	217.79	
	**STD**	**17.36**	**7.26**	**0.14**	**0.50**		**8.65**
Kalamon	Mean	295.91	30.44	21.25	21.86	347.60	

	**STD**	**143.61**	**3.78**	**0.30**	**2.22**		**81.75**
Konservolia	Mean	322.10	28.26	10.38	17.90	360.74	
	**STD**	**85.39**	**7.30**	**0.53**	**7.08**		**47.16**
Koroneiki	Mean	213.13	14.90	3.03	16.84	231.05	
	**STD**	**24.28**	**4.69**	**0.53**	**7.54**		**12.69**
Lastovka	Mean	319.03	20.32	13.52	17.45	352.88	
	**STD**	**125.84**	**0.62**	**3.78**	**6.56**		**71.40**
Leccino	Mean	325.87	29.17	19.46	14.85	374.50	
	**STD**	**94.71**	**12.07**	**14.42**	**0.69**		**47.05**
Leccio del Corno	Mean	192.80	22.33	12.10	14.16	227.23	
	**STD**	**27.87**	**4.58**	**0.42**	**0.12**		**14.79**
Lechin de Sevilla	Mean	184.88	20.09	5.48	17.42	210.45	
	**STD**	**98.79**	**6.07**	**5.27**	**2.78**		**53.76**
Majorca	Mean	139.52	11.64	10.05	16.37	161.21	
	**STD**	**1.80**	**0.57**	**0.22**	**0.17**		**0.83**
Manzanilla de Jaen	Mean	458.58	51.21	5.80	9.10	515.59	
	**STD**	**12.14**	**1.70**	**1.13**	**1.28**		**6.20**
Manzanilla de Sevilla	Mean	214.92	35.67	9.24	16.13	259.83	
	**STD**	**91.36**	**29.21**	**1.79**	**6.08**		**45.90**
Maurino	Mean	307.06	24.40	10.83	15.02	342.28	
	**STD**	**180.35**	**17.26**	**0.46**	**2.45**		**99.36**
Memecik	Mean	326.44	28.63	19.11	18.14	374.19	
	**STD**	**149.24**	**9.18**	**1.40**	**0.83**		**83.20**
Meski	Mean	260.92	44.74	15.57	13.92	321.23	
	**STD**	**180.74**	**48.16**	**9.83**	**0.94**		**89.68**
Moraiolo	Mean	282.44	23.98	11.95	19.88	318.36	
	**STD**	**125.27**	**18.70**	**10.88**	**5.29**		**63.90**
Moresca	Mean	273.64	18.41	13.85	21.19	305.90	
	**STD**	**194.95**	**10.46**	**0.21**	**5.52**		**109.60**
Morisca	Mean	280.74	23.83	14.85	23.02	319.42	
	**STD**	**9.53**	**2.58**	**1.20**	**0.14**		**4.47**
Morrut	Mean	202.05	18.71	10.60	20.64	231.36	
	**STD**	**90.51**	**15.35**	**0.57**	**4.55**		**48.23**
Nocellara del Belice	Mean	262.00	25.70	8.50	16.32	296.20	
	**STD**	**16.97**	**0.57**	**0.14**	**1.27**		**9.60**
Nociara	Mean	373.56	35.64	9.00	16.75	418.20	
	**STD**	**7.76**	**8.50**	**0.14**	**1.88**		**4.63**
Oblica	Mean	218.89	18.83	9.48	18.95	247.20	
	**STD**	**79.05**	**12.30**	**9.24**	**2.38**		**39.45**
Passalunara	Mean	135.77	13.57	6.55	22.07	155.89	
	**STD**	**38.26**	**4.78**	**0.08**	**2.31**		**20.82**
Pendolino	Mean	378.49	23.39	9.33	10.49	411.21	
	**STD**	**239.75**	**4.15**	**4.07**	**0.40**		**136.05**
Peranzana	Mean	390.27	30.86	9.80	16.74	430.93	
	**STD**	**5.66**	**0.94**	**0.42**	**2.84**		**2.88**
Picholine de Languedoc	Mean	291.97	31.45	7.56	18.87	330.98	
	**STD**	**105.03**	**33.43**	**6.64**	**2.77**		**50.87**
Picholine Marocaine	Mean	214.83	18.55	11.85	16.88	245.23	
	**STD**	**107.65**	**2.05**	**2.19**	**3.58**		**60.93**
Picual	Mean	286.11	21.19	10.43	16.82	317.72	
	**STD**	**214.81**	**9.25**	**2.23**	**5.40**		**120.76**
Picudo	Mean	217.76	19.80	7.20	16.65	244.76	
	**STD**	**118.69**	**13.11**	**3.54**	**1.87**		**63.90**
Pizz’e Carroga	Mean	297.16	26.59	12.49	23.32	336.24	

	**STD**	**103.09**	**11.48**	**1.40**	**6.28**		**56.03**
Rowghani	Mean	286.55	46.25	7.25	16.20	340.05	
	**STD**	**4.88**	**1.34**	**1.48**	**0.42**		**2.00**
Salonenque	Mean	236.59	32.29	7.76	18.21	276.63	
	**STD**	**114.56**	**7.18**	**3.17**	**2.89**		**63.18**
Tanche	Mean	302.85	20.57	9.08	19.80	332.51	
	**STD**	**105.07**	**6.68**	**5.12**	**1.88**		**57.26**
Tonda iblea	Mean	179.26	13.68	3.78	18.60	196.71	
	**STD**	**89.03**	**6.55**	**2.44**	**3.38**		**48.85**
Uovo di Piccione	Mean	157.20	18.96	2.30	18.05	178.46	
	**STD**	**10.49**	**4.47**	**0.14**	**3.83**		**5.20**
Vera	Mean	149.05	18.93	5.65	24.23	173.63	
	**STD**	**18.52**	**5.20**	**0.07**	**0.15**		**9.52**
Verdale	Mean	156.00	12.35	3.15	14.77	171.50	
	**STD**	**2.83**	**1.20**	**0.49**	**0.78**		**1.20**
Zaity	Mean	201.81	15.66	6.63	16.38	224.10	
	**STD**	**105.85**	**10.33**	**7.60**	**1.78**		**55.95**
Zard	Mean	377.13	41.06	10.76	18.13	428.95	
	**STD**	**244.44**	**33.75**	**0.06**	**1.50**		**132.45**

* Total tocopherols for each cultivar.

** Total STD is a standard deviation of all tocopherols from each STD.

Table S5. Descriptive statistics by analysis of the nineteen chemical parameters (expressed as mg kg-1 of fruit pulp) in 61 cultivars during two consecutive crop seasons.

Crop season		Minimum	Maximum	Mean	Std. Error	Std. Deviation
2015-2016	HTYR	120.00	3549.00	700.12	64.49	580.45
	TYR	97.00	1571.00	248.28	18.65	167.85
	D_OLEU	54.00	24393.00	2108.86	528.83	4759.51
	OLEU	335.00	36755.00	6873.11	821.90	7397.09
	OLEOC	183.00	3436.00	819.77	70.95	638.57
	VER	4.00	8067.00	840.14	152.58	1373.26
	RUT	120.00	4212.00	1455.70	104.05	936.44
	LUT_7G	50.00	1308.00	430.14	30.74	276.62
	LUE	20.00	593.00	131.81	12.80	115.16
	API_7G	52.00	672.00	261.25	15.05	135.45
	SQU	564.00	5874.00	1799.25	107.32	965.92
	CAMP	5.00	36.00	14.31	0.71	6.41
	ST_STE	1.00	57.00	9.20	0.85	7.63
	β_STE	56.00	530.00	245.94	11.77	105.94
	β_STA	2.00	80.00	25.48	2.20	19.76
	α_TOC	148.00	739.00	355.69	13.57	122.15
	Y_TOC	12.00	92.00	33.00	1.80	16.22
	β_TOC	0.00	44.00	12.21	0.89	7.97
	OCFFW	8.00	30.00	16.16	0.38	3.43
2016-2017	HTYR	67.00	1416.00	309.25	24.84	267.56
	TYR	30.00	1054.00	194.45	13.44	144.72
	D_OLEU	31.00	20682.00	1127.28	273.24	2942.84
	OLEU	387.00	39766.00	9723.17	784.30	8447.20
	OLEOC	28.00	2015.00	474.14	38.21	411.55
	VER	9.00	4878.00	629.31	93.58	1007.84
	RUT	85.00	6124.00	1186.56	87.68	944.32
	LUT_7G	62.00	2386.00	415.53	31.75	341.96
	LUE	10.00	395.00	65.37	5.74	61.86
	API_7G	50.00	556.00	161.39	8.50	91.53
	SQU	230.00	4352.00	1524.19	94.16	1014.08
	CAMP	2.00	71.00	14.51	0.97	10.39
	ST_STE	1.00	51.00	11.45	1.13	12.14
	β_STE	116.00	900.00	325.84	17.60	189.60
	β_STA	0.00	123.00	16.84	2.03	21.84
	α_TOC	76.00	655.00	194.11	7.83	84.37
	Y_TOC	1.00	66.00	17.97	0.98	10.51
	β_TOC	0.00	25.00	8.57	0.52	5.64
	OCFFW	11.00	33.00	19.99	0.39	4.21

Table S6. Tests of between-subjects effects from general linear model for 61 olive cultivars in two consecutive crop seasons.

	Genotype			Environment			Genotype × Environment		
	F	*P*	*η2(%)*	*F*	*P*	*η2(%)*	*F*	*P*	*η2(%)*
HTYR	105.07	< 0.0001	27.74	2094.49	< 0.0001	9.22	87.48	< 0.0001	18.09
TYR	12.58	< 0.0001	24.74	33.89	< 0.0001	1.11	5.38	< 0.0001	8.29
D_OLEU	260.02	< 0.0001	50.29	134.04	< 0.0001	0.43	226.33	< 0.0001	34.29
VER	270.11	< 0.0001	65.17	24.89	< 0.0001	0.10	30.43	< 0.0001	5.75
RUT	90.09	< 0.0001	26.17	161.44	< 0.0001	0.78	18.84	< 0.0001	4.29
LUT_7G	67.54	< 0.0001	22.40	20.20	< 0.0001	0.11	30.21	< 0.0001	7.85
OLEU	49.59	< 0.0001	33.24	116.91	< 0.0001	1.31	18.73	< 0.0001	9.83
API_7G	22.13	< 0.0001	12.91	348.44	< 0.0001	3.39	17.20	< 0.0001	7.86
OLEOC	30.90	< 0.0001	29.53	166.30	< 0.0001	2.65	11.10	< 0.0001	8.31
LUE	26.41	< 0.0001	33.73	182.25	< 0.0001	3.88	8.61	< 0.0001	8.62
SQU	39.76	< 0.0001	14.93	58.88	< 0.0001	0.37	31.80	< 0.0001	9.36
CAMP	10.62	< 0.0001	18.05	0.00	1.00	0.00	4.57	< 0.0001	6.08
ST_STE	7.82	< 0.0001	24.36	12.17	< 0.0001	0.63	7.49	< 0.0001	18.28
β_STE	34.42	< 0.0001	13.59	161.88	< 0.0001	1.07	21.72	< 0.0001	6.72
β_STA	34.62	< 0.0001	38.24	102.01	< 0.0001	1.88	13.13	< 0.0001	11.36
α_TOC	5.90	< 0.0001	7.71	285.84	< 0.0001	6.23	2.33	< 0.0001	2.39
Y_TOC	5.02	< 0.0001	10.43	170.95	< 0.0001	5.92	4.93	< 0.0001	8.02
β_TOC	18.70	< 0.0001	18.86	103.11	< 0.0001	1.73	11.59	< 0.0001	9.16
OCFFW	5.58	< 0.0001	2.57	115.34	< 0.0001	0.89	2.27	< 0.0001	0.82
Mean of *η2(%)*			24.98			2.19			9.76

Eta squared (η2): percentage of trait variability explained by each factor and the interaction. In bold the traits with highest source effect.
F ratio: variation between samples/variation within the samples. p-value: significance level.
